# A comparative study of factors influencing residents' waste sorting behavior in urban and rural areas of China

**DOI:** 10.1016/j.heliyon.2024.e30591

**Published:** 2024-05-01

**Authors:** Shibin Zhang, Yuzi Luo, Pu Zhang

**Affiliations:** School of Management Engineering, Shandong Jianzhu University, Jinan, 250101, China

**Keywords:** Waste sorting behavior, Theory of planned behavior, Norm activation model, Economic incentives, Policy restraints

## Abstract

Extensive research has been conducted on the waste sorting behavior (WSB) of residents, while it is the first time that the classification behavior of urban and rural residents is compared under the same theoretical framework in China. Based on questionnaire data from 478 urban and rural residents, structural equation modeling (SEM) was used to investigate the internal factors influencing the WSB by integrating the Theory of Planned Behavior (TPB) and the Norm Activation Model (NAM). Hierarchical regression analysis was utilized to investigate the moderating effect of external factors on the residents' intentions and behavior. The results show that the degree of deviation between rural residents' intentions and behavior is much larger than that of urban residents. Personal norms are the key factors affecting urban residents' waste sorting. In contrast, for rural residents, attitude is the most critical factor, but the influence of subjective norms is insignificant. In addition, we found that policy restraints and economic incentives significantly moderate the association between urban residents' sorting intention and behavior, with economic incentives having a better effect than policy restraints. In contrast, the impact of policy restraints on rural residents is better than that of urban areas. However, the moderating effect of economic incentives is insignificant for rural residents. The findings furnish the government with meaningful strategies to narrow the urban-rural waste management gap.

## Highlights

• Comparing the sorting behavior of urban and rural residents is the first time.
• Applying the integrated TPB-NAM to the study of residents' waste sorting behavior.
• The moderating effect of economic incentives on rural residents is insignificant.
• Policy restraints are more effective for rural residents than urban residents.

## 1 Introduction

China faces severe waste treatment and management problems as one of the world's largest economies. In 2019, China's urban domestic waste generation was 235 million tons, a 21.9 % increase from 2018, and is projected to exceed 480 million tons by 2030 [1]. About 172 million tons of trash are produced annually in rural areas, with a per capita daily production is 0.8 kg [2], and the dilemma of waste surrounding villages is becoming more and more significant. At present, China's urban waste disposal rate can reach more than 90 %, while the rural waste disposal rate is only about 50 %, while the classification and management of domestic waste is an effective way to achieve waste reduction, resource utilization, and harmlessness [3]. In the face of the rapid growth of waste generation in China, how to categorize and manage the unbalanced development of urban and rural areas is a realistic and urgent issue.

In 2019, China first officially started a pilot waste classification program in Shanghai. Subsequently, Beijing, Tianjin, Jinan, and 46 other key cities were also included in the pilot program and required mandatory waste classification [4]. Shandong Province, China's second most populous and third most economically important province, faces severe resource pressures and environmental problems due to long-term rapid urbanization [5]. Jinan, as the capital of Shandong Province and a pilot city for waste classification, plays a leading role in the demonstration. On May 1, 2021, “Jinan City Domestic Waste Classification, Reduction and Classification Management Regulations” was officially implemented [6].On the one hand, the implementation of the regulations further stipulates the corresponding penalty standards, in which individuals who fail to separate household garbage at designated locations will be fined from 50 yuan to 200 yuan. On the other hand, residents are motivated to separate waste through exemplary creation activities and the introduction of evaluation criteria for “standardized towns”. Besides, it also promotes the formation of good behavioral habits of garbage classification by individuals through a variety of ways, such as points exchange and the gift of classified garbage bags. By 2023, 3260 neighborhoods and 4691 villages in Jinan had been fully covered to carry out garbage classification, and the recycling rate of domestic garbage exceeded 36 %. Domestic waste classification is an important tool for urban governance and ecological civilization in Jinan, yet the impact and comparison of policy implementation on the classification behavior of urban and rural residents remains inconclusive. Due to the existence of the status of unbalanced development between urban and rural areas in China, there are differences in the influencing elements that determine whether urban and rural residents participate in waste sorting. Residents, as the producers of domestic waste, are also the executors of sorting governance, and their behavior affects the effectiveness of waste sorting governance [7]. A fuller comprehension of the constraints influencing these actions is required to enhance the effectiveness of waste sorting and achieve the coordinated development of urban and rural areas. Therefore, to promote China's progress in the field of urban and rural waste management, it is very practical to compare the internal influencing factors that influence residents' involvement in waste sorting in urban and rural areas and evaluate the efficacy of external factor stimulation.

Extensive research has been conducted on the internal elements that influence people's waste-sorting intentions and behavior. Among them, the theory of planned behavior (TPB) and the theory of norm activation (NAM) are widely used [2,[8], [9], [10]]. According to TPB, behavioral attitudes, subjective norms, and perceived behavioral control, all have an impact on a person's purpose [11]. Within the TPB's framework, several scholars have investigated the WSB of residents in different regions of China and concluded that subjective norms, attitudes, and perceived behavioral control all promote the generation of residents' intention to sort waste [4,10,12], but some scholars believe that subjective norms have a negative influence [7]. Numerous research has demonstrated the feasibility of the TPB for predicting WSB [13,14]. However, the TPB considers self-interest maximization the basic principle of individual behavioral decision-making. Therefore, studying residents' sorting behavior from a self-interested perspective alone will inevitably ignore the influence of personal altruistic motives on residents, making it challenging to comprehensively understand residents' WSB. In contrast, the NAM is considered an altruistic theory, highlighting the significance of ethical reasons in determining selfless conduct [13]. Residents' sense of environmental responsibility and psychological perception of environmental pollution significantly increase residents' intention to sort [15], and ascribed responsibility and their interaction with personal norms are essential predictors of residents' environmental behavior [10]. WSB is a kind of self-interested and altruistic pro-environmental behavior. Many researchers have applied the TPB-NAM integration in the field of pro-environmental behaviors of residents [10,16,17]. Therefore, it is more explanatory and persuasive to employ the TPB and NAM integrated model in waste sorting.

Residents' behavior is not only affected by internal factors but also by external factors [[18], [19], [20]]. On the one hand, the effectiveness of government policy restraints strongly influences individuals' environmental protection intentions and behavior. Tian et al. found that residents in areas with waste sorting policies are likelier to implement WSB than those without [21]. Meanwhile, effective policy advocacy can enhance residents' environmental awareness and improve their perception of consequences [9,22,23], which indicates that the influence of external factors can promote change in residents' internal psychological factors, which in turn prompts residents to adopt sorting behavior. In addition, the economic penalties for non-sorting behavior can also force residents to classify waste [6]. On the other hand, many cities have introduced some incentive mechanisms to encourage residents to sort waste actively. For example, Beijing, Shanghai, and other regions have introduced “green points” to exchange rewards. Tianjin has established different levels of demonstration awards for waste classification according to the level of awards. Meanwhile, some waste sorting professional service companies joined in, such as “love classification”, where recyclable items are rewarded with 0.8 yuan per kilogram of “environmental protection gold”. Some scholars believe that groups receiving government subsidies may be more likely to understand the government's waste separation policy, and thus carry out waste separation [24]. However, some studies have shown that economic incentives are ineffective, and direct material incentives even discourage residents' sorting behavior [25]. As a result, the effectiveness of the incentives is inconclusive.

Overall, the current research on Chinese residents' WSB is enriching. However, the following limitations still exist: (1)The majority of research on how urban or rural people sort their waste is done separately. There is a lack of comparative studies on rural and urban residents' participation in waste separation under the same theoretical framework, so it is difficult to see the differences in the implementation of waste separation between urban and rural areas. Considering WSB is a kind of self-interested and altruistic pro-environmental behavior, it is more explanatory and persuasive to employ the TPB and NAM integrated model in waste sorting. (2) Regarding the research on external factors, many scholars analyze external factors as direct factors of intention or behavior, and it is difficult to test the effectiveness of government policy implementation. Considering that there is still a significant difference between the sorting intention and behavior, it is more realistic to explore whether the moderating impact of external factors can narrow the gap between intention and behavior for formulating government policies.

In light of the existing knowledge gap, this study aims to contribute to the current body of knowledge using 478 questionnaires from urban and rural residents of the same region. Firstly, this study aims to investigate the internal factors affecting the WSB of individuals in both urban and rural areas and to test whether the stimulation of external factors can promote the transformation of residents' sorting intention into sorting behavior. Secondly, this paper combines the theories of TPB and NAM and explores the moderating effects of economic incentives and policy restraints in the relationship between residents' intentions and behavior, which enriches the existing theoretical framework. Thirdly, this is the first time to compare the classification behavior of urban and rural residents under the same theoretical framework. The findings of the research can provide valuable insights for the government to formulate policies related to waste management and narrow the gap between the current situation of urban and rural waste management by drawing on the experience of waste management in advanced regions.

The remainder of the structure of the paper is as follows. The theoretical framework and research hypothesis are presented in Section 2. The methodology is covered in Section 3. The results are in Section 4. The discussion and implications are in Section 5, and the conclusion and limitations are in Section 6.

## 2 Theoretical framework and research hypothesis

### 2.1 The theory of planned behavior (TPB)

Ajzen proposed the theory of planned behavior (TPB) to explain and anticipate people's overall behavior [26]. The theory holds that the most reliable variable for predicting an individual's behavior is their intention, and the individual's intention is affected by psychological factors, including subjective norms (SN), behavioral attitudes (ATT), and perceived behavioral control (PBC). First, SN is characterized as a person's impression of societal pressure when making decisions. It's vital in explaining residents' sorting and recycling behavior [27]. The results of previous studies on waste recycling behavior suggest that social influences from friends and family support will encourage each person to recycle their waste [9,28]. Secondly, ATT refers to a behavior or object's positive or negative evaluation. Individuals' pro-environmental attitude is the primary factor in their recycling behaviors [17]. Finally, PBC is the perceived ability to conduct a behavior. The stronger the perception of the ease or difficulty of achieving a behavior, the easier it is for an individual to develop the intention to act, leading to the actual behavior. Research on specific waste recycling behaviors, such as recycling plastic waste [29], recycling construction waste [16,30], and recycling e-waste [17], have shown that SN, ATT, and PBC all positively affect recycling intention. In addition, PBC can also directly influence individual behaviors when the individual can perceive the limitations of the objective conditions. The following hypotheses are thus proposed：

H1 The sorting intention is positively impacted by SN.

H2 The sorting intention is positively impacted by ATT.

H3a The sorting intention is positively influenced by PBC.

H3b The sorting behavior is positively impacted by PBC.

H4 The sorting behavior is positively impacted by the sorting intention.

### 2.2 The norm activation model (NAM)

Schwartz put out the norm activation model (NAM) as an explanation for altruistic conduct [31]. The theory makes use of three variables to explain behavioral intention including personal norms (PN), awareness of the consequences (AC), and ascribed responsibility (AR). First, PN is the antecedent of behavioral intentions, which is the moral obligation to conduct a behavior. Altruistic actions are more common among those with strong PN, as evidenced by previous studies [8,11]. Moreover, PN are internal factors that drive waste recycling [10,32]. Second, AC refers to whether someone realizes that acting in a way that is not socially beneficial can harm other people or things. Individuals' environmental awareness and AC of perceived pollution of the environment from waste will better promote recycling behavior among residents [33]. Furthermore, it is generally accepted that the ability to perceive the consequences of one's actions can facilitate AR [1,11]. Third, AR is the term used to describe taking responsibility for the unfavorable effects of not acting in a way that benefits society. Thus, PN of sorting waste will be activated, and they will tend to classify waste when they are aware of the benefits of sorting waste and feel that they are accountable for socially sustainable growth. The following hypotheses are thus proposed：

H5a AR is positively impacted by AC.

H5b PN is positively impacted by AC.

H6 PN is positively impacted by AR.

H7 The sorting intention is positively impacted by PN.

### 2.3 Integrating TPB and NAM

SN are the rules and standards that residents perceive in their daily lives from the groups around them for implementing WSB, and people tend to be influenced by others to act following the behavior of the majority [10]. Therefore, residents' perceived SN will promote the influence of PN on their waste sorting in the form of social pressure. In addition, residents' ATT toward waste sorting may be influenced by AC. ATT reflects residents' cognition of their participation in waste sorting. In contrast, AC reflects residents' understanding of the impacts of the results of their behaviors, so an individual's cognition of consequences inevitably affects their attitudes toward participation in behaviors [9]. Han found that when individuals perceive the expected negative impacts of lodging on the environment, they hold more positive attitudes toward waste recycling [8]. The following hypotheses are thus proposed：

H8 Residents' PN is positively impacted by SN.

H9 AC positively affects residents' ATT toward waste sorting.

### 2.4 Moderating effect of policy restraints and economic incentives

Although behavioral intentions can predict actual behavior to some extent, a growing number of scholars have found that many residents who self-report good intentions do not produce actual behavior [34], which suggests that there is still a great deal of variability in residents' intention and behavior.

Economic incentives refer to residents paying a specific cost for WSB while receiving corresponding financial compensation and rewards [22]. Participating in waste separation and recycling is a beneficial social behavior that can generate positive environmental externalities. Whereas a suitable environment has a public good attribute, usually without external incentive stimulation, a rational agent seldom or never contributes to the public good because he can still obtain non-excludable mutual gains at the expense of the labor of others. Ji et al. empirically demonstrated that monetary incentives motivate individual recycling behaviors [35]. External economic incentives have great potential to increase willingness and waste separation behavior. Therefore, the hypothesis is proposed.

H10 The association between sorting intention and behavior is positively moderated by economic incentives.

Policy restraints are the government's restrictions and limitations on residents' behaviors at the institutional level to internalize environmental externalities. Government policy restraints are essential in enhancing residents' environmentalist actions [9]. Some research has shown that the effectiveness of government policy implementation promotes individuals' implementation of recycling behaviors [13]. Without policy restraints, many residents will not voluntarily implement sorting behavior even if they have a strong intention to sort. Therefore, policy restraints produce a certain moderating effect between the intention to sort and behavior. The hypothesis is proposed.

H11 The association between sorting intention and behavior is positively moderated by policy restraints.

**Fig. 1:**
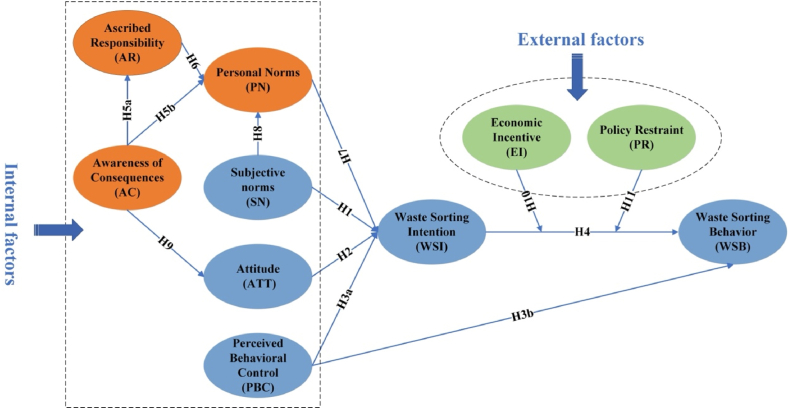
Conceptual framework of the study.

The hypothetical path and theorical framework diagram for this paper is shown in Fig. 1.

## 3 Methodology

### 3.1 Questionnaire design

There were two sections to the questionnaire. The first section investigates the background information of the residents, including their age, gender, level of education, area of residence, and other essential information. The second part is to design questions for the 10 variables involved in the theoretical model of this paper. The scale design refers to the existing mature scales and is measured by a five-point Likert scale [2,[9], [10], [11],^13^,^17^,^22^,^26^,^27^,^31^,^32^,^35^].To assess the residents’ WSB, we may inquire about how frequently they sort their waste. 1 = “never”, 2 = “rarely”, 3 = “occasionally”, 4 = “often”, and 5 = “always”. Policy restraints were measured by how strong the restraints were, numbers 1 through 5 represent “very small,” “relatively small,” “general,” “relatively large,” and “very large,” respectively, and other variables were measured from “strongly disagree (=1)" to “strongly agree (=5)". The measurements were modified to fit the residents' participation in the waste sorting scenario, as shown in Table 1.

**Table 1:** Measurement scales in the formal questionnaire.

Variables		Measurement items	Source
SN	SN1	Relatives and friends around you often take the initiative to sort waste.	[17,26,27]
	SN2	Your relatives and friends around you are in favor of waste separation.	
	SN3	Your relatives and friends want you to sort waste.	
ATT	ATT1	It is a wise choice to sort waste.	
	ATT2	It is the right choice to sort waste.	
	ATT3	It is important to sort waste.	
	ATT4	I support everyone's participation in waste separation.	
PBC	PBC1	I have lots of chances to sort my garbage at home.	
	PBC2	It's convenient for me to sort waste.	
	PBC3	I am aware of which household waste materials are recyclable.	
	PBC4	I am aware of the location where I can recycle my household waste.	
AC	AC1	Failure to separate garbage will result in pollution.	[11,31,32]
	AC2	If we do not separate waste, it will harm the lives of future generations.	
AR	AR1	Improved waste separation is our obligation.	
	AR2	It is everyone's obligation to separate waste.	
	AR3	If I don't separate my household waste, I should be accountable.	
PN	PN1	I think I should take the initiative to participate in waste separation.	
	PN2	I feel it is my responsibility to reduce the pollution of waste separation.	
	PN3	I think we must consider the impact on the environment when littering waste.	
	PN4	My values will encourage me to be more proactive in waste separation.	
	PN5	Throwing away waste makes me feel guilty.	
EI	EI1	Participation in waste separation will be rewarded with certain material incentives, such as cash or gifts.	[22,35]
	EI2	Participation in waste separation will be rewarded with some moral incentives, such as honor or recognition.	
PR	PR1	Frequency of publicity and education on waste separation.	[9,13]
	PR2	The degree of strict supervision of village committees/neighborhood committees for failing to segregate waste.	
	PR3	Severe penalties are imposed for failing to separate waste.	
BI	BI1	I am willing to sort waste.	[2,10]
	BI2	I plan to participate more in waste separation in the future.	
	BI3	I am likely to participate in waste separation in the future.	
	BI4	I will encourage my relatives and friends to participate in waste separation.	
WSB	WSB1	How often do you sort food waste from other waste?	
	WSB2	How often do you sort recyclable waste from other waste?	
	WSB3	How often do you sort hazardous waste from other waste?	
	WSB4	How often do you sort construction trash from other waste?	

Note: SN= Subjective norms, ATT = Attitude, PBC=Perceived behavioral control, AC = aware of consequences, AR=Ascribed responsibility, PN=Personal norms, EI = Economic Incentive, PR=Policy Restraint, BI = behavioral intention, WSB=Waste sorting behavior.

### 3.2 Study area and sample distribution

Jinan, the capital of Shandong Province, is one of the first 46 pilot cities for waste classification in China [4]. It has a population of 9.415 million and lies in the center of Shandong Province, China. The city has 10 districts and 2 counties, with a total area of 10,244.45 square kilometers.

Jinan plays a leading role in a demonstration in the field of waste classification, from 2019 was selected in the country's first batch of 46 pilot cities' list of waste classification, to May 1, 2021, the official implementation of the “Jinan City Domestic Waste Classification, Reduction, and Classification Management Regulations”, Jinan domestic waste classification management into the age of legal basis [6]. By 2022, 3260 neighborhoods and 4691 villages in Jinan had full coverage of waste classification, and the recycling rate of domestic waste exceeded 36 %. Therefore, it is representative and persuasive to use Jinan as a case study to investigate the garbage classification behavior of urban and rural residents

**Fig. 2:**
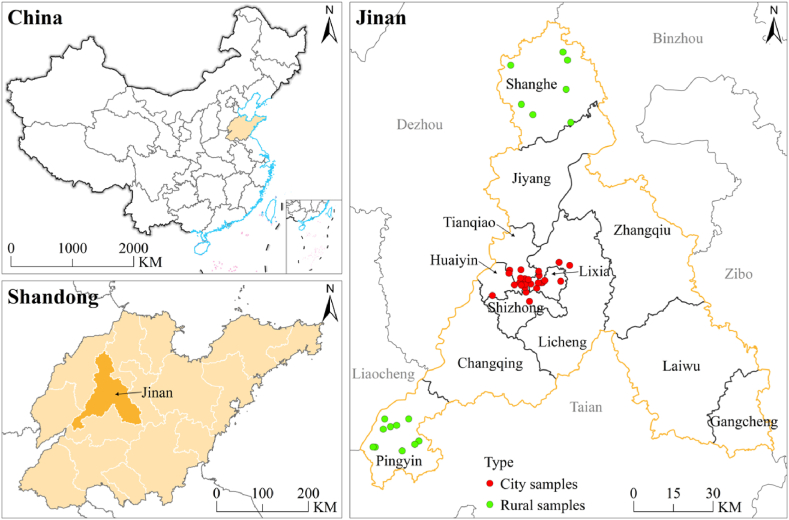
Study area and sample distribution.

The survey was mainly conducted in June–August 2023. The questionnaire of the urban areas is mainly in the center of Jinan City (including Lixia District, Licheng District, Shizhong District, Huaiyin District, and Tianqiao District), and fielded surveys of permanent residents in six to eight different types of communities randomly selected in each district. The questionnaire of the rural areas is mainly in the towns of Donge Town, Kongcun Town, Baiqiao Town, and Longsangsi Town in Pingyin and Shanghe County to obtain questionnaires or one-on-one interviews with local villagers. The specific sample distribution is shown in Fig. 2.

### 3.3 Data collection

This research strictly adhered to the principles outlined in the Helsinki Declaration. All participants provided informed consent before participating in the study. Participants' anonymity and confidentiality were guaranteed, and participation was entirely voluntary. The ethical procedures of this research were reviewed and verified by the School of Management Engineering, Shandong Jianzhu University.

To make the survey samples conform to the actual situation of the urban and rural population, stratified random sampling was used to ensure that each level of the population was considered and that the gender was balanced, with a certain proportion of people of different ages, educational backgrounds, and income levels. At the same time, in order to ensure the quality of the data, part of the survey was conducted through the network for anonymous distribution of questionnaires, and part of the survey used on-site scanning of electronic questionnaires to complete the answer sheet, in addition, for some older or less educated residents, one-on-one interviews were used to record and organize the questionnaires, and professional training was given to the team members, and the results of the questionnaires were audited and qualified to be entered.

A total of 280 electronic questionnaires were distributed through WeChat and QQ, excluding invalid questionnaires such as missing answers and short answer time, 216 valid questionnaires were recovered, with an effective rate of 77.14 %. A total of 200 valid paper questionnaires were distributed and recovered through offline scanning of the questionnaires and on-site distribution of the questionnaires through publicity in the community. In addition, 62 paper questionnaires were collected and organized through one-on-one interviews. The total number of valid questionnaires is 478, including 275 urban questionnaires, accounting for 57.53 %, and 203 rural questionnaires, accounting for 42.47 %. The basic characteristics of the sample of survey respondents are shown in Table 2. From the viewpoint of gender ratio, age distribution, education level, and other indicators, the sample is consistent with the characteristics of the distribution of urban and rural populations in China and is highly representative.

**Table 2:** Descriptive statistical analysis.

Variables	Urban areas	Rural areas
*Gender*		
Male	42.2 %	32.5 %
Female	57.8 %	67.5 %
*Age*		
18–25	24.7.5 %	13.8 %
26–35	24.4 %	9.9 %
36–50	30.2 %	28.1 %
>51	20.7 %	54.2 %
*Education*		
Middle school or below	13.1 %	32 %
High school/Secondary school	22.9 %	39.4 %
Bachelor's degree	53.1 %	27.1 %
Master's degree or above	10.9 %	1.5 %

Many studies have suggested that the same research setting may artificially create covariation between predictor and criterion variables, which is referred to as common method bias (CMB). CMB can occur when research is conducted using a single instrument, particularly self-report measures [36]. Our survey data were obtained through a self-report questionnaire. Therefore, Harmon's single-component analysis is employed to evaluate the existence of CMB. The results revealed that the variable accounted for a maximum of 35 percent of the total variance, which did not exceed the criterion of 50 percent.

## 4 Results

### 4.1 Reliability analysis

The reliability analysis of the questionnaire was first carried out using SPSS 23.0 software, and the results are shown in Table 3. The Cronbach's α coefficients of all the variables are all over 0.70, indicating the scale has good reliability.

**Table 3:** Reliability analysis.

Variables	Items	Cronbach's alpha	
		Urban areas	Rural areas
Subjective norms (SN)	3	0.793	0.868
Attitude (ATT)	4	0.860	0.914
Perceived Behavioral Control (PBC)	4	0.872	0.937
Awareness of Consequences (AC)	2	0.758	0.891
Ascribed Responsibility (AR)	3	0.830	0.905
Personal Norms (PN)	5	0.906	0.926
Economic Incentive (EI)	2	0.739	0.863
Policy Restraint (PR)	3	0.932	0.918
Behavioral Intention (BI)	4	0.896	0.938
Waste Sorting Behavior (WSB)	4	0.907	0.883

### 4.2 Validity analysis

Next, the questionnaire was tested for validity, which refers to the accuracy and validity of the content of the measurement results. First, the content of the questionnaire in this study was designed based on mature scales and reviewed by experts and scholars, so the content validity is acceptable.

Second, Amos 26.0 was used to conduct a validated factor analysis (CFA) of the questionnaire in rural and urban areas, respectively. A measurement model containing 34 observed variables and 10 latent variables was constructed, and the standardized factor loadings of each observed variable were greater than 0.5 [16], indicating that the measurement items corresponding to each latent variable were highly representative. According to the results of the questionnaire fit index in urban areas, CMIN/DF = 2.601 (<3), RMSEA = 0.077 (<0.08), GFI = 0.862, CFI = 0.914, and IFI = 0.94, which are all greater than 0.8. The results of the questionnaire fit index in rural areas showed that CMIN/DF = 2.300 (<3), RMSEA = 0.06 (<0.08), GFI = 0.888, CFI = 0.904, IFI = 0.905, all greater than 0.8. All the fitted indicators in the two regions satisfy the basic requirements, suggesting that the overall model fits well.

Third, the convergent validity of the scale was tested. Convergent validity refers to the degree of convergence of indicators measuring the same concept [37]. The main indicators for verifying the convergent validity are the combined reliability CR and the mean-variance of variance AVE. As seen in Table 4, the mean-variance of variance AVE of each latent variable in urban and rural areas is greater than 0.5, and the combined reliability C.R. value is greater than 0.7, indicating that the scale has good convergent validity.

**Table 4:** Convergent validity analysis.

Variables	Items	Urban areas			Rural areas		
		Estimate	AVE	C.R.	Estimate	AVE	C.R.
SN	SN1	0.595	0.586	0.805	0.723	0.709	0.879
	SN2	0.768			0.852		
	SN3	0.903			0.938		
ATT	ATT1	0.774	0.620	0.866	0.775	0.747	0.921
	ATT2	0.894			0.932		
	ATT3	0.746			0.953		
	ATT4	0.725			0.781		
PBC	PBC1	0.770	0.643	0.878	0.907	0.789	0.937
	PBC2	0.891			0.906		
	PBC3	0.752			0.864		
	PBC4	0.788			0.876		
AC	AC1	0.828	0.620	0.765	0.875	0.804	0.891
	AC2	0.745			0.918		
AR	AR1	0.864	0.664	0.853	0.894	0.768	0.908
	AR2	0.909			0.895		
	AR3	0.648			0.838		
PN	PN1	0.846	0.665	0.908	0.867	0.716	0.926
	PN2	0.861			0.744		
	PN3	0.837			0.938		
	PN4	0.842			0.956		
	PN5	0.676			0.695		
EI	EI1	0.752	0.595	0.746	0.834	0.773	0.872
	EI2	0.790			0.922		
PR	PR1	0.871	0.826	0.934	0.908	0.798	0.922
	PR2	0.962			0.953		
	PR3	0.891			0.813		
BI	BI1	0.770	0.688	0.898	0.868	0.793	0.939
	BI2	0.875			0.893		
	BI3	0.845			0.921		
	BI4	0.824			0.879		
WSB	WSB1	0.827	0.711	0.90s8	0.918	0.649	0.879
	WSB2	0.837			0.710		
	WSB3	0.825			0.889		
	WSB4	0.882			0.677		

Finally, the discriminant validity of the scale was tested. Distinguishing validity refers to the degree of difference between multiple variables. The absolute value of the correlation coefficient between variables is less than the square root of the corresponding AVE, indicating some correlation between the latent variables and some discriminant validity between them [38]. As can be seen in Table 5, except for the non-significant relationship between subjective norms and behavioral attitudes in urban areas, and in Table 6, perceived behavioral control and economic incentives, and policy restraints and economic incentives in rural areas, there are significant correlations between the variables, and the values of the correlation coefficients are less than the square root of the AVE, so the discriminative validity of the data from the scales is good.

**Table 5:** Discriminative validity test of urban areas.

Variables	SN	ATT	PBC	AC	AR	PN	EI	PR	BI	WSB
SN	**(0.766)**									
ATT	0.081	**(0.787)**								
PBC	0.445***	0.107***	**(0.802)**							
AC	0.176***	0.205***	0.313***	**(0.840)**						
AR	0.184***	0.240***	0.374***	0.621***	**(0.865)**					
PN	0.235***	0.246***	0.393***	0.701***	0.752***	**(0.869)**				
EI	0.184***	0.112**	0.153**	0.153**	0.198***	0.248***	**(0.837)**			
PR	0.095***	0.121**	0.119**	0.146**	0.178***	0.301***	0.271***	**(0.879)**		
BI	0.274***	0.268***	0.292***	0.440***	0.441***	0.586***	0.453***	0.342***	**(0.892)**	
WSB	0.280***	0.013	0.289***	0.220**	0.221***	0.322**	0.602***	0.483***	0.529***	**(0.749)**

Note: **and *** represent the statistical significance at 5 % and 1 %, respectively. In parentheses is the average extract square root of variance (AVE).

**Table 6:** Discriminative validity test of rural areas.

Variables	SN	ATT	PBC	AC	AR	PN	EI	PR	BI	WSB
SN	**(0.766)**									
ATT	0.510***	**(0.806)**								
PBC	0.645***	0.555***	**(0.835)**							
AC	0.472***	0.621***	0.553***	**(0.766)**						
AR	0.459***	0.561***	0.582***	0.795***	**(0.865)**					
PN	0.368***	0.391***	0.331***	0.480***	0.507***	**(0.869)**				
EI	0.162**	0.315***	0.128	0.275***	0.219***	0.157**	**(0.837)**			
PR	0.580***	0.305***	0.758***	0.436***	0.499***	0.280***	0.112	**(0.879)**		
BI	0.451***	0.634***	0.495***	0.490***	0.464***	0.464***	0.218***	0.315***	**(0.892)**	
WSB	0.316***	0.189***	0.285***	0.175**	0.146**	0.113**	0.154**	0.306***	0.213***	**(0.749)**

Note: **and *** represent the statistical significance at 5 % and 1 %, respectively. In parentheses is the average extract square root of variance (AVE).

### 4.3 Hypothesis testing analysis

The proposed integrated model was tested using hypothesis testing analysis following the validity and reliability analyses. Before the study hypothesis was put to the test, the model's fit was confirmed. The results of the model fit in urban areas showed that CMIN/DF = 2.44 (<3), RMSEA = 0.072(<0.08), GFI = 0.889(>0.8), CFI = 0.907(>0.9), and IFI = 0.908(>0.9). The results of model fit in rural areas showed that CMIN/DF = 2.308 (<3), RMSEA = 0.053 (<0.08), GFI = 0.893(>0.8), CFI = 0.953(>0.9), IFI = 0.954(>0.9). All the fitting indexes satisfy the basic requirements, which indicates that the structural models in urban and rural areas are well-fitted.

The mean value of urban residents' waste sorting intention is 4.37. The mean value of sorting behavior is 4.03, while the mean value of rural residents' intention is 4.19. The mean value of sorting behavior is 2.58, which indicates a contradiction between their intention and behavior in both regions. The degree of contradiction between rural residents' intention and their behavior is much larger than that of urban residents.

**Fig. 3:**
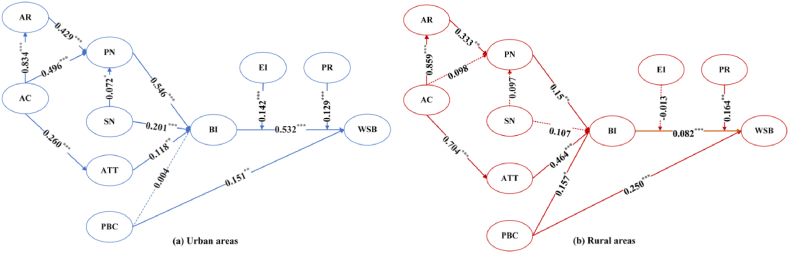
Hypothetical results in urban areas & rural areas.

The theoretical framework results are shown in Table 7 and Fig. 3. In urban areas, SN, ATT, and PN significantly influenced BI, thus verifying hypotheses H1, H2, and H7. The relationship between PBC and BI is insignificant, whereas PBC significantly influenced WSB, thus rejecting H3a and supporting H3b. As expected, BI positively promoted WSB, and H4 is supported. The study revealed that AR and SN significantly influenced PN. Thus, H6 and H8 are supported. The outcomes also show that AC had a positive impact on AR, PN, and ATT. Thus, hypotheses H5a, H5b, and H9 are supported.

**Table 7:** Hypothesis test results.

Hypothesis	Paths	Urban areas				Rural areas			
		Estimate	S.E.	C.R.	Support	Estimate	S.E.	C.R.	Support
H1	SN→ BI	0.201***	0.043	3.105	YES	0.107	0.078	1.248	NO
H2	ATT→ BI	0.118**	0.073	2.121	YES	0.464***	0.081	6.900	YES
H3a	PBC→ BI	0.004	0.057	0.067	NO	0.157*	0.078	1.811	YES
H3b	PBC→ WSB	0.151**	0.057	2.488	YES	0.250***	0.095	2.915	YES
H4	BI→ WSB	0.532***	0.070	8.052	YES	0.082***	0.075	2.969	YES
H5a	AC→ AR	0.834***	0.103	8.889	YES	0.859***	0.059	13.595	YES
H5b	AC→ PN	0.496***	0.116	4.290	YES	0.098	0.191	0.603	NO
H6	AR→ PN	0.429***	0.100	8.889	YES	0.333**	0.193	2.183	YES
H7	PN→ BI	0.546***	0.078	8.342	YES	0.150**	0.062	2.448	YES
H8	SN→ PN	0.072*	0.023	1.772	YES	0.097	0.077	1.144	NO
H9	AC→ ATT	0.260***	0.065	3.694	YES	0.704***	0.060	11.576	YES

Note: The statistical significance at 10 %, 5 %, and 1 %, respectively, is shown by the symbols *, **, and ***.

SN= Subjective norms, ATT = Attitude, PBC=Perceived behavioral control, AC = aware of consequences, PN=Personal norms, AR=Ascribed responsibility, BI = behavioral intention, WSB=Waste sorting behavior.

In rural areas. ATT, PBC, and PN significantly influenced BI and thus verified the hypothesis H2, H3a, and H7. PBC and BI can directly promote WSB. Therefore, H3b and H4 are supported. The outcomes also show that AC had a positive impact on AR and ATT. AR had a beneficial impact on PN. These findings support hypotheses H5a, H6, and H9. The conclusions show that AC and SN had no significant effect on PN, which rejected H5b and H8. In addition, SN had no significant impact on BI. Thus, H1 is rejected.

### 4.4 Moderating effects analysis

The moderating effects of economic incentives and policy restraints between urban and rural residents' sorting intention and behavior are investigated using the hierarchical regression analysis.

First, taking WSB as the dependent variable, model 1 with behavioral intention (BI) and economic incentives (EI) as the independent variables, and model 2 with the addition of the interaction term of behavioral intention and economic incentives (BI*EI) are constructed, respectively. The moderating impact is significant if the interaction term's regression coefficient passes the significance test [39]. From Table 8, EI significantly moderates the correlation between BI and WSB among urban residents. In contrast, it does not have a moderating impact on the rural residents. Thus, hypothesis H10 is supported in urban areas, however, the hypothesis is rejected in rural areas. The same approach is used to construct models 3 and model 4 on policy restraints (PR). The results are shown in Table 9, which show that PR can significantly and positively regulate the correlation between BI and WSB in urban and rural areas. Therefore, H11 is supported in urban and rural areas.

**Table 8:** The moderating effect of economic incentive.

	Model 1				Model 2			
	Urban areas		Rural areas		Urban areas		Rural areas	
	Estimate	T-value	Estimate	T-value	Estimate	T-value	Estimate	T-value
Cons	0.356***	6.968	1.275***	3.374	0.632***	5.432	1.280***	3.370
BI	0.323***	6.370	0.188***	2.675	0.355***	6.967	0.187***	2.635
EI	0.456***	9.014	0.113	1.608	0.462***	9.267	0.114	1.613
BI*EI					0.142***	3.091	−0.013	−0.181
R^2^	0.446		0.048		0.464		0.043	
F value	109.311		6.096		78.351		4.055	

Note: The statistical significance at 10 %, 5 %, and 1 %, respectively, is shown by the symbols *, **, and ***.

Dependent variable: WSB=Waste sorting behavior, BI = behavioral intention, EI = economic incentives.

**Table 9:** The moderating effect of policy restraint.

	Model 3				Model 4			
	Urban areas		Rural areas		Urban areas		Rural areas	
	Estimate	T-value	Estimate	T-value	Estimate	T-value	Estimate	T-value
Cons	0.973***	3.981	1.166***	3.442	0.816***	3.276	1.065***	3.160
BI	0.414***	8.153	0.129*	1.838	0.447***	8.640	0.136*	1.958
PR	0.339***	6.678	0.265***	3.766	0.328***	6.508	0.271***	3.903
BI*PR					0.129***	2.644	0.164**	2.478
R^2^	0.383		0.108		0.399		0.135	
F value	84.438		12.169		59.883		10.368	

Note: The statistical significance at 10 %, 5 %, and 1 %, respectively, is shown by the symbols *, **, and ***.

Dependent variable: WSB=Waste sorting behavior, BI = behavioral intention, PR = policy restraints.

To explain the moderating effect of EI and PR more intuitively, the moderating impact diagram is drawn on this basis.

**Fig. 4:**
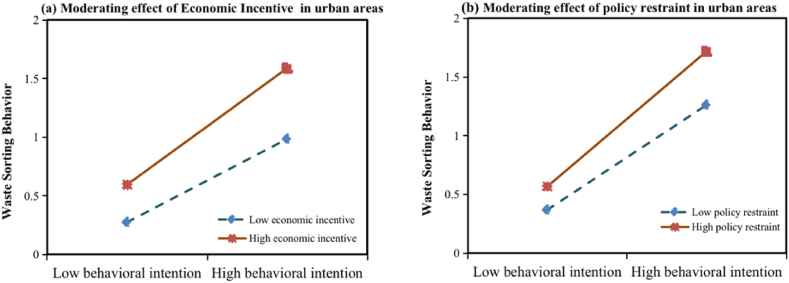
Moderating effect of policy restraint & economic incentive in urban areas.

Fig. 4 shows the moderating effects of EI and PR on urban residents' BI and WSB, which shows that the higher the level of PR and the stronger the EI, the higher the consistency between BI and WSB (with greater slopes). In contrast, when PR is less strong and the EI is weaker, the lower the consistency (with lower slopes). The moderating effect value of PR in urban areas is 0.129 (p < 0.001), and the value of EI is 0.142 (p < 0.001), indicating that EI works better than PR in urban areas.

**Fig. 5:**
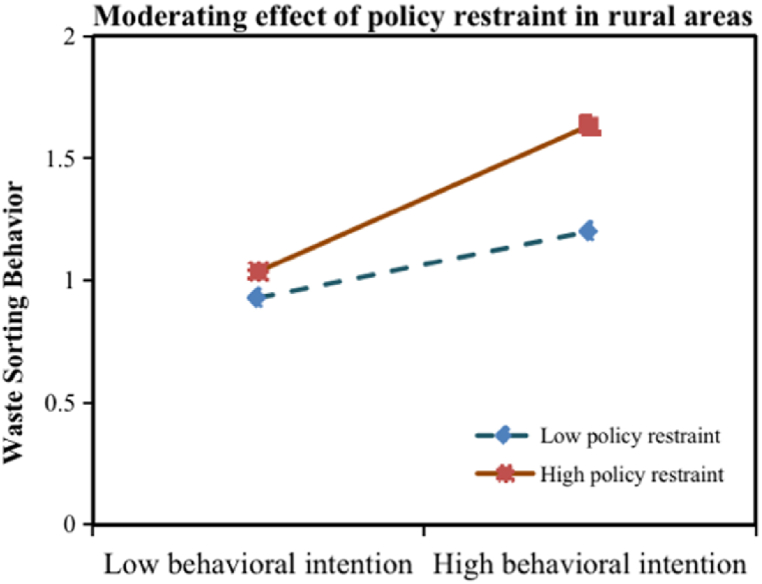
Moderating effect of policy restraint in rural areas.

Fig. 5 demonstrates the moderating effect of PR on rural residents' BI and WSB. We can see that the slope of the straight line is relatively steep under high PR, indicating that the intention to sort strongly influences sorting behavior. The slope of the straight line is relatively slower under low PR, indicating that the positive effect of the intention on sorting behavior is gradually weakening. Therefore, the stricter the degree of PR, the more likely it is that people in rural areas will sort waste. The value of the moderating effect of PR in rural areas is 0.164 (p < 0.001), which is higher than that in urban areas (β = 0.129, p < 0.001), indicating that PR is more effective in rural areas.

## 5 Discussion and implications

### 5.1 Discussion

The research demonstrates a deviation between residents' waste sorting intention and behavior in urban and rural areas in China, and the intention-behavior deviation is greater in rural than urban areas. The empirical analysis yields that intention significantly contributes to the emergence of behavior both in urban and rural areas, but the promotion effect for urban residents (β = 0.532, p < 0.001) is much more significant than for rural residents (β = 0.082, p < 0.001).

Personal norms (β = 0.546, p < 0.001) are the most crucial factor influencing urban residents' waste sorting, while the main factor influencing rural residents is attitude (β = 0.464, p < 0.001). This indicates that the main internal factor for urban residents to sort waste is altruistic motivation. In contrast, rural residents are more focused on self-interested motivation. This may be due to the fact that residents in urban areas are generally more educated and have already deeply realized that failure to classify waste will cause pollution to the environment and infringe on the health of themselves and others, so they will constrain their personal norms to sort waste actively and proactively. However, rural populations have an inferior degree of education, and the sorting behavior is mainly due to personal attitudes. Given the properties of the environment as a public good, there is widespread “free-riding behavior” among rural residents [9]. Residents who approach waste sorting with a positive attitude will do, while those with an indifferent or wait-and-see attitude rarely implement waste sorting behaviors.

As expected, subjective norms significantly and positively influenced urban residents' personal norms and behavioral intentions, but in rural areas, the role of subjective norms was insignificant. This finding reveals that a good atmosphere for waste separation has been formed in urban areas. However, rural areas are still far behind. The possible reason for this is that due to the early stage of waste separation pilots were mainly carried out in urban areas, and the participation rate of residents in rural areas was low [27], which has not yet been able to form an intense atmosphere of separation.

The correlation between awareness of consequences and the other three factors (ascribed responsibility, attitudes, and personal norms) was significant for urban residents. In contrast, the correlation between awareness of consequences and personal norms was insignificant for rural residents. This implies that urban residents are highly educated and have strong environmental awareness and a more vital perception of environmental pollution caused by failure to segregate waste [1,11]. Residents' intention to separate will be influenced by personal norms that are stimulated by their awareness of the consequences and their ascribed responsibility for waste separation. Furthermore, it may be because even if rural residents understand that littering will have negative consequences for the environment since they benefit from the convenience of littering in the past. Their behaviors are reinforced, and they cannot easily step out of their comfort zones [32]. The awareness of consequences has not risen to the level of personal norms, and only by stimulating internal motivation can rural residents be motivated to sort waste.

An interesting finding is that the perceived behavioral control of urban residents doesn't significantly influence their sorting intention, while it directly promotes sorting behavior, whereas perceived behavioral control of rural residents promotes their intention and behavior significantly. Prior studies have shown that facility accessibility, behavioral habits, and past experiences affect people's perceived behavioral control, which in turn affects their behavior [2,40,41]. Compared to rural areas, urban waste separation infrastructure is more complete, and facility accessibility directly promotes WSB. Due to facility accessibility and daily behavioral habits, many urban residents do not regard the difficulty of waste separation as a problem, and as long as residents have sufficient environmental awareness and a strong sense of responsibility for waste recycling, they will ignore the perceived control of waste separation and implement recycling behaviors [9]. Some scholars have shown in empirical studies that an individual's intention towards a behavior is more likely to be generated by personal attitudes and subjective norms than perceived behavioral control [42,43]. Thus, many residents in urban areas do not plan to sort their garbage before discarding it, but when they encounter the ubiquitous sorting bins by the side of the road, driven by personal attitudes and norms drive them to sort their garbage temporarily. In rural areas, it is more difficult to separate garbage probably because of the lack of accessibility and the lack of habit of separating garbage.

The positive moderating effect of policy restraints is significant on urban and rural residents' intentions and behavior, and policy restraints are more effective in rural areas than in urban areas. Most cities in China, including Jinan, have made it plain that people who illegally dispose of domestic waste would face consequences. Specifically, they have stated that violators will pay a fine of not less than 50 yuan but not more than 200 yuan. Prior research has demonstrated that WSB can be successfully encouraged by legislative restrictions [21,22]. Rural residents have stronger perceived economic losses and will be more willing to restrain their behavior to avoid unnecessary economic losses [24]. In urban areas, many residents have formed daily habits of waste separation and do not need to rely on too many external restraints. In contrast, some external restraints are required for rural residents to stimulate their behavior. Hence, government publicity and some punitive measures, such as fines for failure to carry out waste separation, are more effective in promoting WSB among rural residents.

The positive moderating impact of economic incentives on residents' intention and behavior is significant in urban areas but not in rural areas. Currently, in many communities that set up recycling sites in Jinan, residents can instantly receive incentives feedback, such as cash prizes, gift redemption, etc., and the government will also select the garbage classification model communities and towns, to enhance the residents' sense of honor. We generally believe that incentives can reduce waste separation's time and economic costs to influence their behavior [35]. Most residents in urban areas have already enjoyed the economic benefits brought by sorting waste and believe that they can obtain specific economic and material incentives while protecting the environment. The possibility of transforming the sorting intention into actual behaviors has subsequently increased. However, the implementation of incentives for waste separation has not yet been fully covered in rural areas, and most villagers have yet to enjoy the honors and incentives that come with the implementation of waste separation. Therefore, economic incentives cannot stimulate the altruistic mindset of rural residents in the short term due to the solidification of behaviors and perceptions, so their initiative to implement waste separation behaviors is also relatively weak.

### 5.2 Policy implication

Most waste management participants are citizens of both urban and rural areas, and the unity of their behavioral intention and behavior is crucial to promoting the development of waste management in China and realizing the construction of waste-free cities. Consequently, considering the distinctions between urban and rural locations, and the policy audience's differences and demand preferences, the following policy insights are obtained.

#### 5.2.1 Policy implication in urban areas

First, personal norms have the most significant influence on urban residents' behavioral intentions, and they have a strong awareness of consequences. To further encourage residents to participate in waste sorting, the government should organize and conduct educational lectures and themed activities at various levels to stimulate residents' sense of responsibility to protect the environment by participating in waste sorting. Second, subjective norms positively influence urban residents' behavioral intentions. The government should formulate practical guidelines and standards for waste sorting, and continuously promote them to strengthen the residents' sense of identity and responsibility to classify waste and to form a good atmosphere and culture of waste sorting in the whole society. Third, to promote the transformation of urban residents' intentions into behavior, the government is recommended to guide and encourage urban residents to join in the formulation, implementation, and supervision of incentive policies directly or indirectly for waste sorting so that residents can be more aware of relevant material rewards and other incentives for waste sorting, thus mobilizing the enthusiasm of residents to sort waste. Furthermore, although some cities have established restraints on waste sorting, they lack an effective monitoring mechanism. Policymakers are advised to establish a grid-based supervision and management system based on the district or community as a unit in urban areas.

#### 5.2.2 Policy implication in rural areas

First, although attitudes are the main factor influencing rural residents' waste sorting, attitudes have not risen to the level of personal norms, and a good atmosphere for waste sorting has not been formed in rural areas. Therefore, rural areas can learn from urban areas, and rural residents' personal norms can be stimulated by the awareness of consequences and ascribed responsibility to generate internal drives to participate in waste sorting proactively. Second, considering the lower level of education of rural residents, the environmental deterioration caused by unclassified waste can be presented through pictures and videos to strengthen the ecological cognitive ability of the residents in rural areas, stimulate their sense of responsibility, and enhance their intention to sort waste. Third, policy restraints can significantly transform rural residents' intentions into behavior. The government should strengthen the top-level system design, increase personnel and financial investment, and smooth the feedback and supervision path for residents. For example, a grid-based supervision mechanism can be established. In rural areas, a farmer with strong environmental awareness can be voted by farmers' organizations to serve as a grid leader. It is also possible for the government to elect a prestigious farmer among old party members, rural elites, village capable persons, and model workers to oversee the waste sorting work to ensure the policy's consistency and stability. Moreover, policymakers are advised to provide cash rewards to residents who correctly sort waste and cash punishment to those who do not carry out waste sorting to make the implementation of the policy more effective.

## 6 Conclusion and limitations

### 6.1 Conclusion

Based on the data from 478 questionnaires, integrating the theory of TPB and NAM, SEM was used to understand the internal factors affecting urban and rural residents' WSB, and hierarchical regression analysis was utilized to explore the moderating effects of the external factors of economic incentives and policy restraints between residents' behavioral intention and their sorting behaviors. The main conclusions are as follows:

First, both urban and rural residents' behavioral intention can promote their WSB, but there is a deviation between their intention and behavior, and the degree of deviation in rural areas is much larger than that in urban areas.

Second, personal norms are the most critical factor influencing urban residents' WSB. In contrast, the correlation between perceived behavioral control and urban residents' behavioral intention is insignificant. Economic incentives and policy restraints positively regulate the correlation between intention and behavior, and the effect of economic incentives is better than that of policy restraints.

Third, attitude mainly affects rural residents' WSB. In contrast, the impact of subjective norms is insignificant, and the correlation between awareness of consequences and personal norms is insignificant. Policy restraints positively regulate the relation between intention and behavior, and the effect of policy restraints is better than that of urban areas, while the regulatory impact of economic incentives is not significant.

### 6.2 Limitations

Although our research concludes with some interesting findings and insights, there are limitations. Firstly, domestic waste sorting and recycling treatment is a systematic work. There are more factors affecting the effect of sorting behavior. The variables selected in this paper are only a part of them, and the subsequent research can incorporate more influencing factors into the framework for research. Secondly, the paper is based on the cross-section data of the questionnaire, which cannot dynamically react to the impact of the stimulation of external factors on the behavior of waste sorting. In the future, we can use the way of constructing simulation to simulate the effects of implementing economic incentives and policy restraints.

## Funding

The study was funded by Shandong Province Natural Science Foundation (ZR2020QG062) and Social Science Planning Project of Shandong Province (20CGLJ18).

## CRediT authorship contribution statement

**Shibin Zhang:** Conceptualization, Funding acquisition, Writing – review & editing. **Yuzi Luo:** Data curation, Investigation, Methodology, Writing – original draft, Writing – review & editing. **Pu Zhang:** Supervision, Visualization, Writing – original draft, Writing – review & editing.

## Declaration of competing interest

The authors declare that they have no known competing financial interests or personal relationships that could have appeared to influence the work reported in this paper.
