# Supplementary material for: A comparative study of factors influencing residents' waste sorting behavior in urban and rural areas of China
Source: Heliyon. 2024 May 1;10(9):e30591. doi: 10.1016/j.heliyon.2024.e30591 (PMC11096745; doi:10.1016/j.heliyon.2024.e30591)
Supplement: Multimedia component 1 [file mmc1.docx]

**Informed Consent**

Dear participants

Thank you very much for participating in our survey. This questionnaire is a study on the willingness and behavior of residents to participate in waste separation. Your answers are very important to our research, so please answer truthfully based on your actual perceptions and behaviors.

This participation is purely voluntary, and you may terminate your participation in this study at any time. The decision will not result in any adverse consequences. This questionnaire is anonymous, and all data are only for academic research and analysis.

The collected information is strictly confidential. If you have any inquiries about this study in the future, you can send an email to yuzi_luo@163.com

If you already understand the above content and are willing to participate in this study, please check “Yes” below.

Yes No

**Body of the questionnaire**

1. What is your gender?
2. Male
3. B. Female
4. What is your age?

A．18-25 years

B. 26-35 years

C. 36-50 years

D. 51-60 years

1. What is your academic qualification?
2. Junior high school and below
3. senior high school/technical school
4. university college
5. bachelor’s degree and above.
6. Are you living in a rural or urban area?

A．rural area

B. urban area

1. Relatives and friends around you often take the initiative to sort waste.
2. Not at all
3. Comparative non-compliance
4. Uncertain
5. Comparative compliance
6. Full compliance
7. Your relatives and friends around you are in favor of waste separation.

A. Not at all

B. Comparative non-compliance

C. Uncertain

D. Comparative compliance

E. Full compliance

1. Your relatives and friends want you to sort waste.

A. Not at all

B. Comparative non-compliance

C. Uncertain

D. Comparative compliance

E. Full compliance

1. It is a wise choice to sort waste.

A. Strongly disagree

B. Relatively disagree

C. General

D. Relatively agree

E. Strongly agree

1. It is the right choice to sort waste.

A. Strongly disagree

B. Relatively disagree

C. General

D. Relatively agree

E. Strongly agree

1. It is important to sort waste.

A. Strongly disagree

B. Relatively disagree

C. General

D. Relatively agree

E. Strongly agree

1. I support everyone's participation in waste separation.

A. Strongly disagree

B. Relatively disagree

C. General

D. Relatively agree

E. Strongly agree

1. I have lots of chances to sort my garbage at home.

A. Strongly disagree

B. Relatively disagree

C. General

D. Relatively agree

E. Strongly agree\

1. It's convenient for me to sort waste.

A. Strongly disagree

B. Relatively disagree

C. General

D. Relatively agree

E. Strongly agree

1. I am aware of which household waste materials are recyclable.

A. Strongly disagree

B. Relatively disagree

C. General

D. Relatively agree

E. Strongly agree

1. I am aware of the location where I can recycle my household waste.

A. Strongly disagree

B. Relatively disagree

C. General

D. Relatively agree

E. Strongly agree

1. Failure to separate garbage will result in pollution.

A. Strongly disagree

B. Relatively disagree

C. General

D. Relatively agree

E. Strongly agree

1. If we do not separate waste, it will harm the lives of future generations.

A. Strongly disagree

B. Relatively disagree

C. General

D. Relatively agree

E. Strongly agree

1. Improved waste separation is our obligation.

A. Strongly disagree

B. Relatively disagree

C. General

D. Relatively agree

E. Strongly agree

1. It is everyone's obligation to separate waste.

A. Strongly disagree

B. Relatively disagree

C. General

D. Relatively agree

E. Strongly agree

1. If I don't separate my household waste, I should be accountable.

A. Strongly disagree

B. Relatively disagree

C. General

D. Relatively agree

E. Strongly agree

1. I think I should take the initiative to participate in waste separation.

A. Strongly disagree

B. Relatively disagree

C. General

D. Relatively agree

E. Strongly agree

1. I feel it is my responsibility to reduce the pollution of waste separation.

A. Strongly disagree

B. Relatively disagree

C. General

D. Relatively agree

E. Strongly agree

1. I think we must consider the impact on the environment when littering waste.

A. Strongly disagree

B. Relatively disagree

C. General

D. Relatively agree

E. Strongly agree

1. My values will encourage me to be more proactive in waste separation.

A. Strongly disagree

B. Relatively disagree

C. General

D. Relatively agree

E. Strongly agree

1. Throwing away waste makes me feel guilty.

A. Strongly disagree

B. Relatively disagree

C. General

D. Relatively agree

E. Strongly agree

1. Participation in waste separation will be rewarded with certain material incentives, such as cash or gifts.

A. Never

B. Hardly

C. Sometimes

D. Often

E. Usually

1. Participation in waste separation will be rewarded with some moral incentives, such as honor or recognition.

A. Never

B. Hardly

C. Sometimes

D. Often

E. Usually

1. Frequency of publicity and education on waste separation.
2. Never
3. Hardly
4. Sometimes
5. Often
6. Usually
7. The degree of strict supervision of village committees/neighborhood committees for failing to segregate waste.
8. Very small
9. Relatively small
10. General
11. Relatively large
12. Very large
13. Severe penalties are imposed for failing to separate waste.

A. Very small

B. Relatively small

C. General

D. Relatively large

E. Very large

1. I am willing to sort waste.

A. Strongly disagree

B. Relatively disagree

C. General

D. Relatively agree

E. Strongly agree

1. I plan to participate more in waste separation in the future.

A. Strongly disagree

B. Relatively disagree

C. General

D. Relatively agree

E. Strongly agree

1. I am likely to participate in waste separation in the future.

A. Strongly disagree

B. Relatively disagree

C. General

D. Relatively agree

E. Strongly agree

1. I will encourage my relatives and friends to participate in waste separation.

A. Strongly disagree

B. Relatively disagree

C. General

D. Relatively agree

E. Strongly agree

1. How often do you sort food waste from other waste?

A. Never

B. Hardly

C. Sometimes

D. Often

E. Usually

1. How often do you sort recyclable waste from other waste?

A. Never

B. Hardly

C. Sometimes

D. Often

E. Usually

1. How often do you sort hazardous waste from other waste?

A. Never

B. Hardly

C. Sometimes

D. Often

E. Usually

1. How often do you sort construction trash from other waste?

A. Never

B. Hardly

C. Sometimes

D. Often

E. Usually
